# The transcription factor CfHac1 regulates the degradation of ubiquitin-mediated ER-associated misfolded proteins and pathogenicity in *Colletotrichum fructicola*

**DOI:** 10.1007/s44154-025-00237-6

**Published:** 2025-06-12

**Authors:** Sizheng Li, Yuan Guo, Shengpei Zhang, He Li

**Affiliations:** 1 State Key Laboratory of Utilization of Woody Oil Resource, Central South University of Forestry and Technology, Changsha, 410004 China; 2 Key Laboratory for Non-Wood Forest Cultivation and Conservation of Ministry of Education, Changsha, 410004 China

**Keywords:** *CfHAC1* gene, Unconventional splicing, Ubiquitination, ER-associated misfolded protein, Pathogenicity

## Abstract

During interactions, pathogenic fungi are subjected to endoplasmic reticulum (ER) stress from the host plants, resulting in the activation of the unfolded protein response (UPR) pathway. We identified the bZIP transcription factor CfHac1 in *C. fructicola*, which is a pathogenic organism implicated in a variety of plant diseases, and we found it to be crucial for the ER stress response and pathogenicity. However, the role of CfHac1 in regulating the degradation of ER-associated misfolded proteins remains unclear. In this study, we discovered that the *CfHAC1* gene regulates conidial production, appressorium formation, response to ER stress, and pathogenicity through unconventional splicing. Further research revealed that the *CfHAC1* gene also affects the ubiquitination of ER-associated misfolded proteins and mediates their degradation. We further identified two ubiquitin ligase genes, *CfHRD1* and *CfHRD3*, that exhibit significant down-regulation in the Δ*Cfhac1* mutant strain. Subsequent investigations revealed that the *CfHAC1* gene affects *CfHRD1* and *CfHRD3* expression through unconventional splicing, with both genes managing the degradation of ER-associated misfolded proteins via ubiquitination and influencing *C. fructicola* pathogenicity. Taken together, our results reveal a mechanism by which the transcription factor CfHac1 affects the expression of the ubiquitin ligase genes *CfHRD1* and *CfHRD3*, leading to the ubiquitination and degradation of ER-associated misfolded proteins and pathogenicity. This provides a theoretical basis for the development of novel agents targeting key genes within this pathway.

**Supplementary Information:**

The online version contains supplementary material available at 10.1007/s44154-025-00237-6.

## Introduction

The genus *Colletotrichum* is acknowledged as one of the ten most significant groups of plant pathogenic fungi worldwide. Numerous species, and occasionally entire genera, have been designated by various countries as critical quarantine targets upon entry. This genus is responsible for anthracnose diseases in agricultural and forestry crops, as well as post-harvest decay in fruits, resulting in substantial economic losses. Additionally, certain species function as opportunistic pathogens, causing keratitis and subcutaneous infections in humans (Dean et al. 2012).

*Colletotrichum fructicola* is a pathogen associated with a range of plant diseases. Initially identified on Coffea in Thailand, it has since been detected on *Pyrus pyrifolia* in Japan, *Limonium sinense* in Israel, *Vitis vinifera* and *Fragaria ananassa* in the United States, *Ficus* in Germany, and tea plants in China (Weir et al. 2012). The geographical distribution of *C. fructicola* is notably extensive, with documented occurrences across five continents, including the Americas, West Asia, East Asia, Western Europe, West Africa, and Australia (Prihastuti et al. 2009; Weir et al. 2012). In previous studies, *C. fructicola* was identified as the principal causal agent of anthracnose in *Camellia oleifera*. Several genes, such as *CfGCN5*, *CfMSG5*, *CfRAD6*, *CfVAM7*, and *CfVPS39*, have been demonstrated to play critical roles in the regulation of pathogenicity (Li et al. 2021a, 2021b; Zhang et al. 2021, 2022; Luo et al. 2023; Gao et al. 2024).

Hac1 is a critical transcription factor that mediates the UPR during endoplasmic reticulum (ER) stress, which occurs due to the accumulation of unfolded proteins in the ER. This pathway is essential for maintaining cellular homeostasis and ensuring proper protein folding and secretion (Fordyce et al. 2012). Current research on the bZIP transcription factor Hac1 primarily focuses on its role in regulating the UPR process (Cox and Walter 1996; Mori et al. 1998; Feng et al. 2011; Mori et al. 1998; Qian et al. 2022; Tang et al. 2015). In the cytosol, inositol-requiring enzyme 1 (Ire1) recruits *HAC1* mRNA, mediating its non-conventional splicing (Anken et al. 2014). Furthermore, the IRE1-mediated splicing of *HAC1* mRNA is a key regulatory step in the UPR, linking it to broader stress responses in various organisms (Zhang et al. 2015). This multifaceted role of Hac1 underscores the significance of bZIP transcription factors in orchestrating cellular responses to environmental challenges. Recently, Yin et al. (2020) reported the presence of ER stress during *M. oryzae* infection, indicating that pathogenic fungi are subjected to ER stress when infecting host plants. However, it is unclear whether Hac1 regulates the degradation of ER-associated misfolded proteins. We identified a homologous bZIP transcription factor, CfHac1, within the genome of *C. fructicola*. We conducted an investigation into the biological functions of CfHac1, determining its essential role in growth, conidiation, appressorium formation, pathogenicity, and the endoplasmic reticulum stress response (Yao et al. 2019; Li et al. 2021a, b).

The ER plays a crucial role in the post-translational modification and folding of secretory and membrane proteins within cells. Upon detection of misfolded proteins, the ER initiates a signaling cascade to the nucleus, prompting the activation of genes aimed at rectifying these folding errors; this cellular mechanism is collectively termed the unfolded protein response (UPR). The endoplasmic reticulum-associated protein degradation (ERAD) pathway exports improperly folded substrates from the ER for degradation by the cytoplasmic ubiquitin–proteasome system, serving as a critical response to ER stress (Smith et al. 2011). The accumulation of unfolded or misfolded proteins results in stress, after which the ERAD pathway transports improperly folded protein substrates from the endoplasmic reticulum for degradation by the cytoplasmic ubiquitin–proteasome system (Smith et al. 2011). The ubiquitin–proteasome system (UPS) is a mechanism present in eukaryotic cells that precisely regulates the orderly degradation of proteins within the cytoplasm and nucleus. It comprises ubiquitin (Ub), ubiquitin-activating enzyme (E1), ubiquitin-conjugating enzyme (E2), ubiquitin ligase (E3), the 26S proteasome, and deubiquitinating enzymes. Ubiquitin molecules are initially activated by E1 and then transferred to E2, with E3 catalyzing the transfer of ubiquitin from E2 to the protein substrate. The main E3 ubiquitin ligases associated with the endoplasmic reticulum are Hrd1 and Doa10, which are primarily responsible for the ubiquitination of substrates.

The current research on the bZIP transcription factor Hac1 mainly focuses on its role in regulating the UPR process, while its regulation of endoplasm reticulum-associated degradation of misfolded proteins remains unclear. In this study, we discovered that *CfHAC1* and its unconventional splicing are crucial for the degradation of ER-associated misfolded proteins. Under ER stress, the *CfHAC1* generates an active transcription factor via unconventional splicing, which enters the nucleus to regulate the expression of the E3 ubiquitin ligase genes *CfHRD1* and *CfHRD3*. This process further ubiquitinates ER-associated misfolded proteins, mediating their degradation, and alleviating cellular stress. Our findings reveal a mechanism by which the transcription factor CfHac1 in *C. fructicola* responds to ER stress and regulates pathogenicity, providing a theoretical basis for the development of novel agents targeting key genes in this pathway.

## Results

### *CfHAC1* undergoes unconventional splicing under endoplasmic reticulum stress

**Fig. 1:**
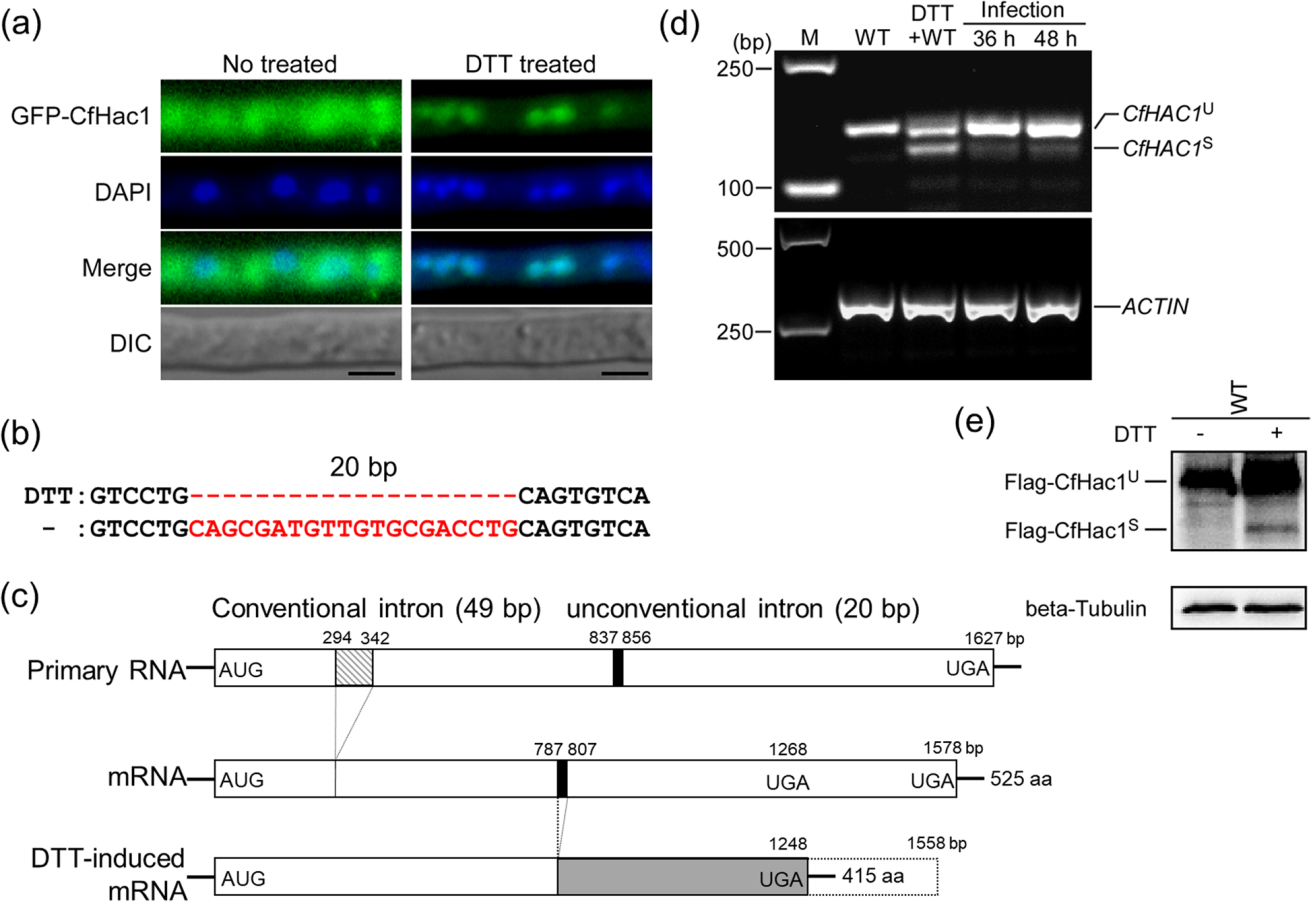
*CfHAC1* mRNA undergoes unconventional splicing under endoplasmic reticulum stress. **a** GFP-CfHac1 fusion proteins were generated as fusions of GFP to the N-terminus of CfHac1. The Δ*Cfhac1* mutants were transformed with a plasmid expressing GFP-CfHac1. The transformed mycelia were treated with 10 mM DTT for 1 h, stained with DAPI, and observed for the subcellular localization of green fluorescence. Scale bar: 5 μm. **b** DNA sequence alignment of the *CfHAC1* (treated with DTT) and *CfHAC1* (treated without DTT) gene. The 20 bp atypical intron is indicated in red font. **c** The diagonal box and solid black box represent the conventional and unconventional intron respectively. The C-terminal fragments of *CfHAC1* before and after unconventional splicing are shown as the white box and a greyed box respectively. **d** RT-PCR was performed using total RNAs extracted from the wild-type strain grown in PD for 2 d, then treated with 10 mM DTT for another 1 h and harvested. For RNA extraction during infection stages, 5 ml of conidial suspension (1 × 10^6^ spores/ml) of CFLH16 were sprayed on 1-week-old *Camellia oleifera* leaves and the infected leaves were collected and cooled with liquid nitrogen. The *ACTIN* gene was used as the control. **e** Western blot of Flag-CfHac1^u^ and Flag-CfHac1^s^

Previous research has indicated that the *CfHAC1* gene is involved in regulating the response of *C. fructicola* to endoplasmic reticulum stress (Yao et al. 2019). To elucidate the molecular mechanism of *CfHAC1* in response to endoplasmic reticulum stress, a fusion expression plasmid of green fluorescent protein (GFP) at the N-terminus of *CfHAC1* was constructed and transformed into the Δ*Cfhac1* strain. We investigated the subcellular localization of CfHac1 under dithiothreitol (DTT) treatment. The results demonstrated that, in the absence of DTT, GFP::CfHac1 green fluorescence was distributed throughout the mycelial cells; however, after DTT treatment, the green fluorescence exhibited aggregation within the mycelial cells. Staining with the nuclear dye 4', 6-diamidino-2-phenylindole (DAPI) revealed that the aggregated green fluorescence overlapped with the blue fluorescence marking the nucleus (Fig. 1a). This indicates that, under DTT treatment, GFP::CfHac1 translocates into the nucleus to regulate the expression of related genes in response to endoplasmic reticulum stress.

TA cloning and sequencing results revealed that, upon exposure to the endoplasmic reticulum stress inducer DTT, the *CfHAC1* gene in the wild-type strain of *C. fructicola* produced two different mRNA transcripts through unconventional splicing: *CfHAC1*^*U*^ and *CfHAC1*^*S*^. Compared to the wild-type strain without stress, the *CfHAC1*^*S*^ transcript was additionally present. Analysis showed that *CfHAC1*^*S*^ was missing a 20 bp nucleotide sequence compared to *CfHAC1*^U^ (Figure S1). These results suggest that, under endoplasmic reticulum stress, the *CfHAC1* mRNA undergoes unconventional splicing, whereby, in addition to the conventional 49 bp intron, a 20 bp unconventional intron is also spliced, resulting in a frameshift mutation in the coding region (Fig. 1b). This frameshift leads to the premature appearance of a stop codon, generating a protein comprising only 415 amino acids, which is 110 amino acids shorter than the protein encoded by the unspliced transcript (Fig. 1c). These results indicate that, in response to endoplasmic reticulum stress, the *CfHAC1* gene in *C. fructicola* undergoes unconventional splicing.

We investigated the splicing of the *CfHAC1* gene under conditions of ER stress. Our findings demonstrated that the wild-type strain of *C. fructicola*, when treated with the ER stress inducer DTT, exhibited an unconventional splicing band that was marginally smaller than the anticipated fragment, as determined by *HAC1*−54F/*HAC1*−55R primers (Fig. 1d). Similarly, the use of these primers revealed a slightly smaller unconventional splicing band in the wild-type strain infecting onion (Fig. 1d). The western blot analysis substantiated the presence of two distinct proteins generated through conventional and unconventional splicing of the *CfHAC1* gene (Fig. 1e). These observations indicate the occurrence of ER stress during *C. fructicola* infection.

### Mutation of the unconventional splicing site of* CfHAC1*

To further elucidate whether the unconventional splicing of the *CfHAC1* gene is associated with the response to endoplasmic reticulum stress, point mutations were introduced into the 20 bp unconventional splicing region. Eight base mutations were designed for the 20 bp unconventional splicing site utilizing codon degeneracy (Figure S2a, b). By employing G418 resistance, homologous recombination was performed to replace the hygromycin B resistance gene in the *CfHAC1* knockout mutant Δ*Cfhac1*. This resulted in the isolation of a point mutant strain Δ*Cfhac1/CfHAC1*^*mut intron*^, which was unable to undergo unconventional splicing of *CfHAC1* mRNA. Using the same in situ repair method, a mutant strain Δ*Cfhac1/CfHAC1*^*S*^, which lacked the 20 bp unconventional splicing region, was also isolated (Figure S2c). Following RNA extraction from the mutant strains and reverse transcription, RT-PCR was conducted using primers *HAC1*−54F/*HAC1*−55R for validation. The results indicated that both mutated genes were effectively expressed in the strains (Figure S2 d). After the exogenous addition of the endoplasmic reticulum stress inducer DTT, only the unspliced *CfHAC1*^*U*^ band was detected in the mutant strain Δ*Cfhac1/CfHAC1*^*mut intron*^, with no unconventional splicing band *CfHAC1*^S^ observed (Figure S2 d). This finding demonstrates that, under endoplasmic reticulum stress, the *CfHAC1*^*mut intron*^ in the mutant strain did not undergo unconventional splicing. Furthermore, in both the presence and absence of DTT, the strain Δ*Cfhac1/CfHAC1*^S^ displayed only the unconventional splicing band *CfHAC1*^S^, with no detection of the *CfHAC1*^*mut intron*^ band (Figure S2 d). These results indicate that point mutations have successfully generated the *CfHAC1* mutant strain Δ*Cfhac1/CfHAC1*^*mut intron*^, which is unable to undergo unconventional splicing, as well as the mutant strain Δ*Cfhac1/CfHAC1*^S^, which lacks the unconventional intron, thereby providing strains for further research.

### The unconventional splicing of the *CfHAC1* is involved in regulating hyphal growth and the response to ER stress

**Fig. 2:**
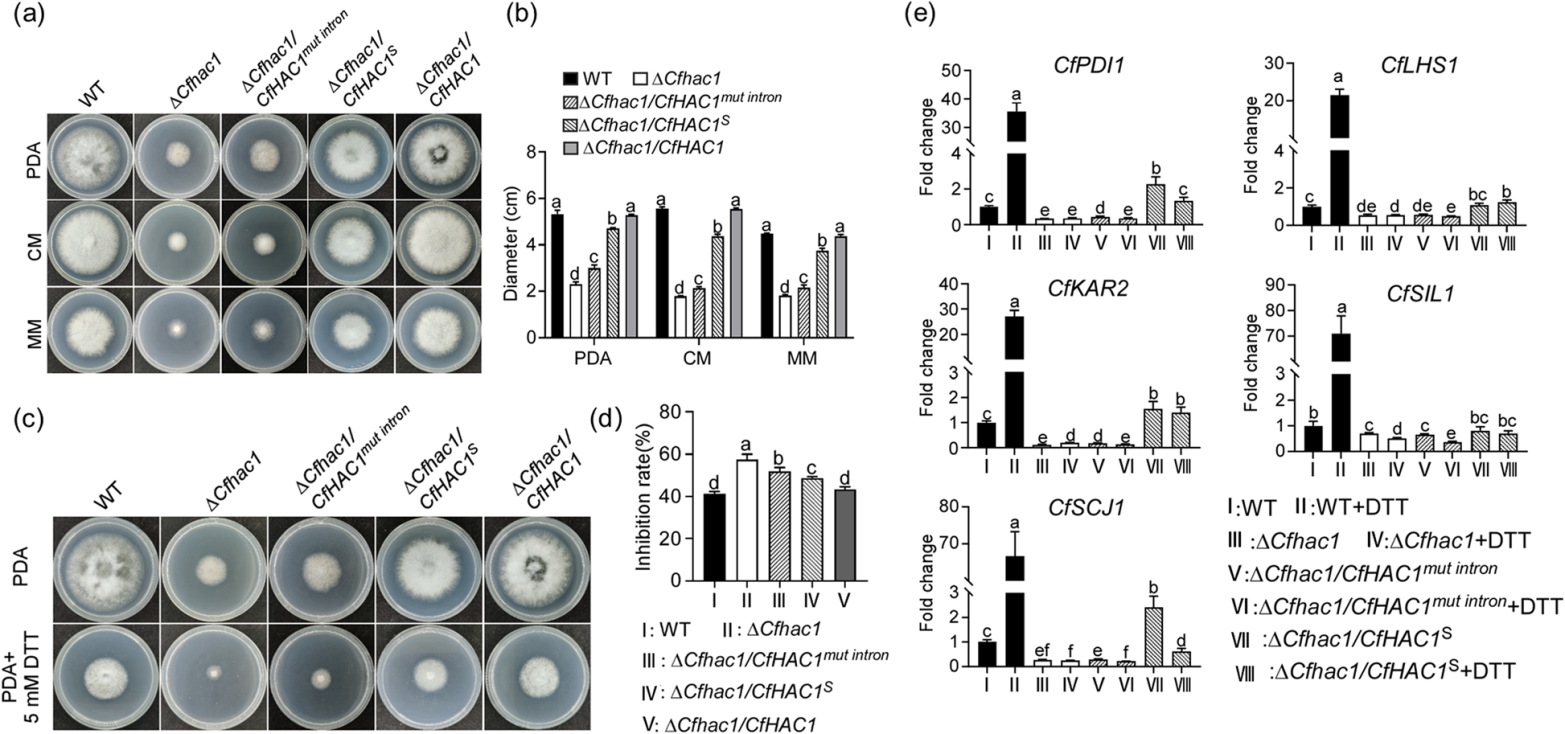
The *CfHAC1* gene knockout mutants and strains defective in unconventional splicing cannot respond to endoplasmic reticulum stress. **a** WT, Δ*Cfhac1*, Δ*Cfhac1/CfHAC1*^*mut intron*^, and Δ*Cfhac1/CfHAC1*^*S*^ were inoculated on PDA, CM, and MM plates, respectively, and cultured at 28 °C in the dark for 6 days. **b** Statistical analysis of the colony diameter was performed. **c** Δ*Cfhac1*, Δ*Cfhac1/CfHAC1*^*mut intron*^, and Δ*Cfhac1/CfHAC1*^*S*^ were inoculated on PDA plates containing 5 mM DTT; **d** Statistical analysis was conducted on the inhibition rates of the strains in relation to endoplasmic reticulum stress. **e** The relative expression levels of putative UPR-regulated genes (*CfPDI1*, *CfLHS1*, *CfKAR2*, *CfSIL1*, and *CfSCJ1*) were analyzed by qRT-PCR and normalized to that of *CfACTIN*. Error bars represent ± SD, and different lowercase letters represent significant differences

To elucidate the role of normal unconventional splicing of the *CfHAC1* gene in strain growth, the growth of colonies of the wild type, Δ*Cfhac1*, Δ*Cfhac1/CfHAC1*^*mut intron*^, and Δ*Cfhac1/CfHAC1*^S^ was compared on PDA, CM, and MM culture media. The results indicated that both Δ*Cfhac1/CfHAC1*^*mut intron*^ and Δ*Cfhac1/CfHAC1*^S^ strains exhibited very sparse aerial mycelium. Although the colony diameter of these two strains was significantly larger than that of the mutant Δ*Cfhac1*, it was still significantly smaller than that of the wild type and the complemented strains (Fig. 2a, b). This finding suggests that the inability to undergo unconventional splicing, or the absence of the 20 bp unconventional splicing region in the *CfHAC1*, could not completely restore the growth defect of Δ*Cfhac1*, indicating that normal unconventional splicing of the *CfHAC1* is essential for strain growth. Furthermore, the colony diameters of Δ*Cfhac1/CfHAC1*^*mut intron*^ and Δ*Cfhac1/CfHAC1*^*S*^ were significantly greater than that of the fully knockout mutant Δ*Cfhac1*, demonstrating that the non-unconventional splicing *CfHAC1*^*mut intron*^ gene and the *CfHAC1*^*S*^ gene, which lacks the 20 bp unconventional splicing region, could partially restore the growth of the Δ*Cfhac1* (Fig. 2a, b).

To determine whether the unconventional splicing of the *CfHAC1* is essential for the response to endoplasmic reticulum stress, the wild type, Δ*Cfhac1* mutant, unconventional splicing mutant strains Δ*Cfhac1/CfHAC1*^*mut intron*^ and Δ*Cfhac1/CfHAC1*^*S*^, as well as the fully complemented strain Δ*Cfhac1/CfHAC1*, were inoculated onto PDA containing 5 mM of the endoplasmic reticulum stress inducer DTT for culture, and colony diameters were measured to analyze the inhibition rate. The results showed that the three mutant strains exhibited slower growth on the DTT-containing medium (Fig. 2c). Statistical analysis revealed that the inhibition rates of the fully mutant strain Δ*Cfhac1*, the point mutant strain Δ*Cfhac1/CfHAC1*^*mut intron*^, and Δ*Cfhac1/CfHAC1*^S^ were significantly greater than those of the wild type and the complemented strains (Fig. 2d). At the same time, we also found that the expression of several putative UPR-target genes, including *CfPDI1*, *CfLHS1*, *CfKAR2*, *CfSIL1*, and *CfSCJ1*, was all increased (Fig. 2e). This indicates that the *CfHAC1* and its ability to undergo unconventional splicing are crucial for the response to ER stress.

### The *CfHAC1* regulates conidiation, appressorium formation, and pathogenicity

**Fig. 3:**
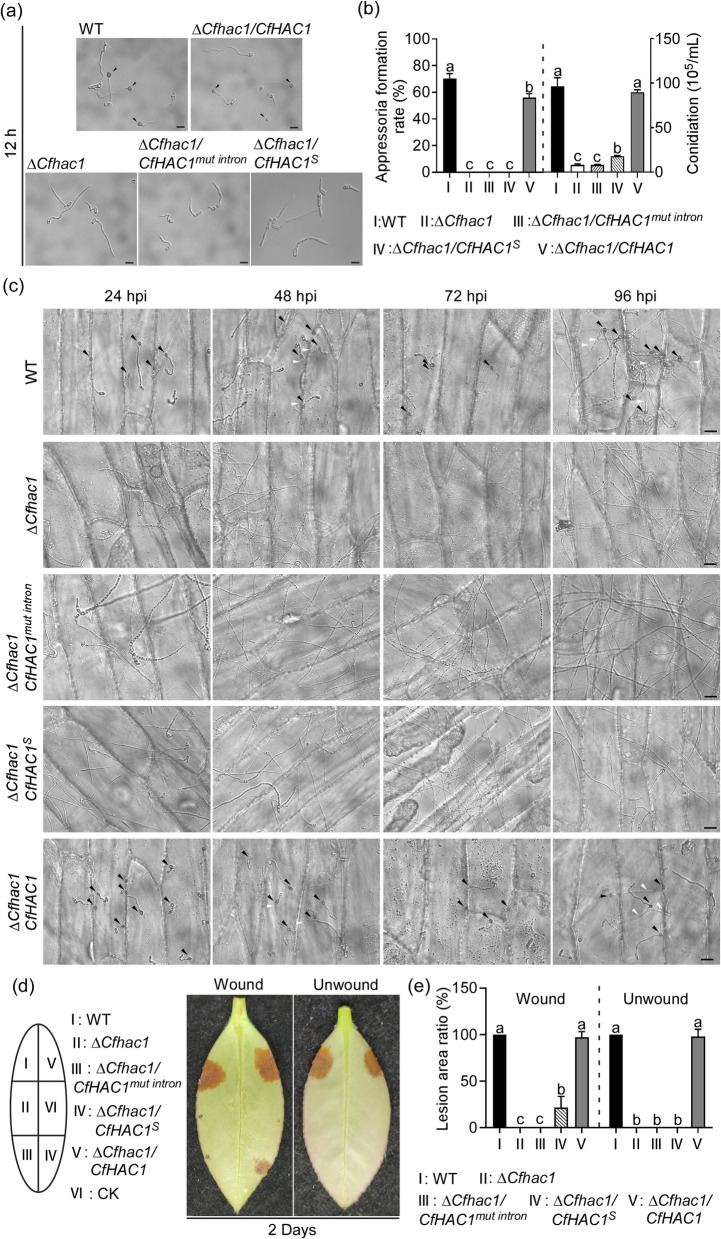
The *CfHAC1* gene regulates pathogenicity. **a** The conidia of WT, Δ*Cfhac1*, Δ*Cfhac1/CfHAC1*^*mut intron*^, Δ*Cfhac1/CfHAC1*^*S*^, and Δ*Cfhac1/CfHAC1*were inoculated on artificial hydrophobic surfaces, respectively. Appressoria formation were observed after inoculation on hydrophobic surfaces at 24 h. Bar = 5 µm. **b** Statistical analysis of appressoria formation rates and conidia production was conducted. The experiments were repeated three times with three replicates, and more than 100 conidia were counted per replicate. **c** The formation of appressoria by the three mutants on onion epidermis. **d** Diseased symptoms of wounded *Camellia oleifera* leaves inoculated with related mycelial blocks at 28℃ for 2 days. **e** Statistical analysis of the ratio of lesion areas of different strains. Error bars represent ± SD, and different lowercase letters represent significant differences

To elucidate whether the pathogenicity of the pathogen requires the normal unconventional splicing of the *CfHAC1*, we studied the production of conidia, appressorium formation, and pathogenicity in the wild type, the Δ*Cfhac*1 mutant, unconventional splicing mutant strains Δ*Cfhac1/CfHAC1*^*mut intron*^ and Δ*Cfhac1/CfHAC1*^*S*^, as well as the fully complemented strain Δ*Cfhac1/CfHAC1*. Compared to the wild type, the three mutants produced fewer conidia and failed to form appressoria (Fig. 3a, b, c), defects restored by the complemented strain Δ*Cfhac1/CfHAC1*; further inoculation assays on onion epidermis over 96 h confirmed their appressorium-deficient phenotype due to impaired hyphal growth. Pathogenicity assays showed that, on wounded *Ca. oleifera* leaves, the Δ*Cfhac1* mutant and the non-unconventional splicing mutant Δ*Cfhac1/CfHAC1*^*mut intron*^ were unable to produce lesions. Only the mutant Δ*Cfhac1/CfHAC1*^*S*^, which lacked the 20 bp unconventional splicing region, formed smaller lesions. On unwounded *Ca. oleifera* leaves, all three mutant strains were incapable of producing lesions(Fig. 3d, e). In contrast, both the wild type and the complemented strain Δ*Cfhac1/CfHAC1* were able to produce large, typical lesions on either wounded or unwounded tea leaves. These results suggest that the *CfHAC1* gene regulates conidial production, appressorium formation, and pathogenicity through unconventional splicing.

### The *CfHAC1* regulates the degradation of ER-associated misfolded proteins

After proteins in organisms have completed their roles or if there are synthesis errors, they often need to be broken down to recycle the amino acids. This process is usually carried out by the ubiquitin–proteasome system. Nonetheless, in certain physiological or pathological situations, autophagy can break down misfolded proteins, protein clumps, and organelles via lysosomal pathways. MG-132 is a cell-permeable proteasome inhibitor that effectively blocks the proteolytic activity of the 26S proteasome complex (Wang et al. 2014). 3-Methyladenine (3-MA), a phosphoinositide 3-kinase (PI3 K) inhibitor, is widely used as an autophagy inhibitor by inhibiting class III PI3 K (Kang et al. 2021). The carboxypeptidase Y mutant CPY* is the most commonly used model protein for studying endoplasmic reticulum-associated degradation (Hiller et al. 1996; Vashistha et al. 2016).

**Fig. 4:**
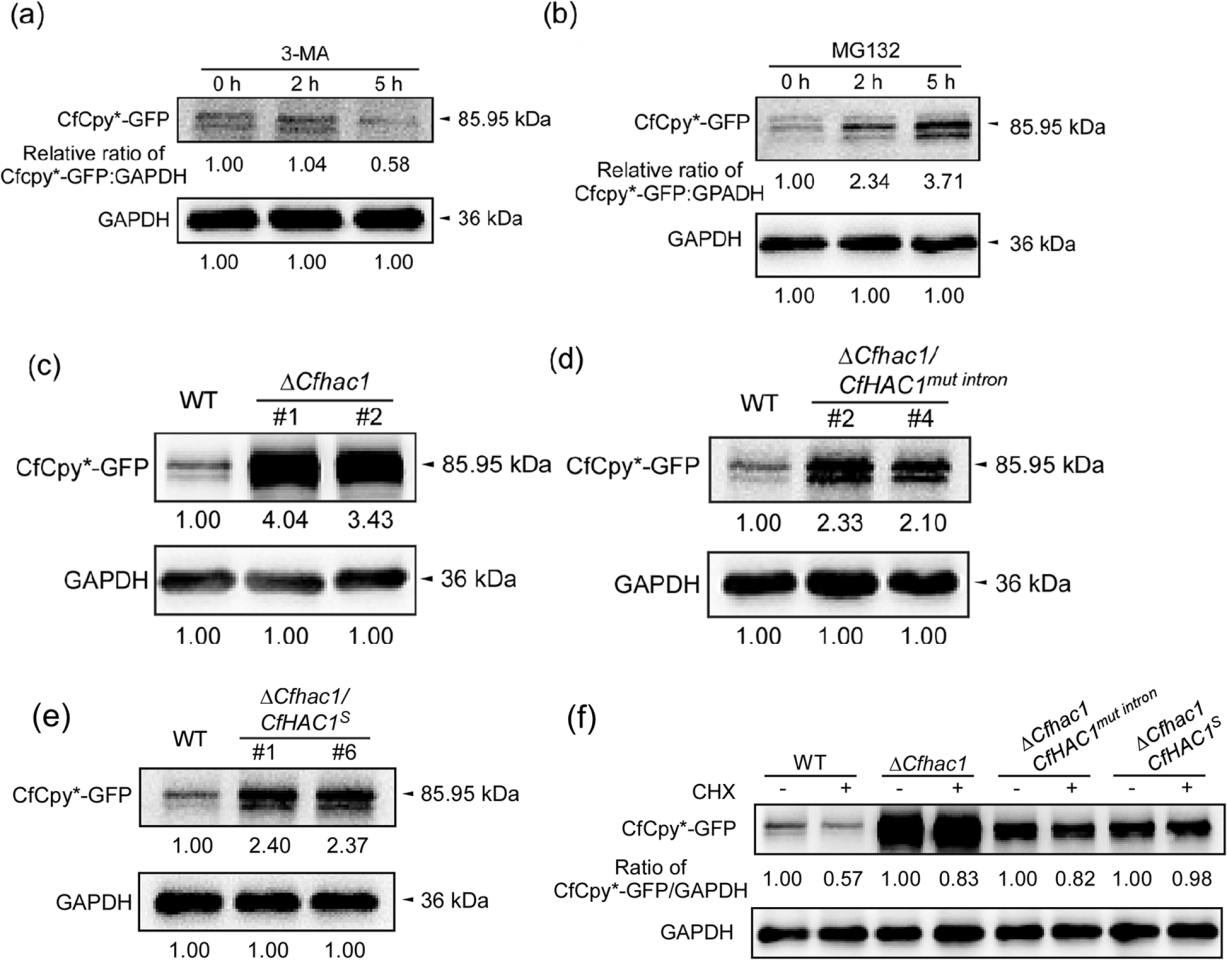
*CfHAC1* modulates the degradation of CfCpy*-GFP. **a** 3-MA was added to PD for 0, 2, and 5 h, after which WT strain mycelia with CfCpy*-GFP were collected, lysed, and analyzed for CfCpy*-GFP degradation using immunoblotting. **b** MG132 was similarly added to PD for 0, 2, and 5 h, and WT strain mycelia with CfCpy*-GFP were processed to assess CfCpy*-GFP degradation via immunoblotting. **c** Degradation of CfCpy*-GFP in the *CfHAC1* gene deletion mutant Δ*Cfhac1*. **d** Degradation of CfCpy*-GFP in the defective unconventional splicing mutant Δ*Cfhac1/CfHAC1*^*mut intron*^. **e** Degradation of CfCpy*-GFP in Δ*Cfhac1/CfHAC1*^*S*^. **f** Degradation of CfCpy*-GFP in the three mutants following cycloheximide (CHX) treatment. GAPDH was used as the control

To determine the degradation pathway of the misfolded protein CfCpy*-GFP, we treated wild-type strains expressing the CfCpy*-GFP fusion protein with both MG-132 and 3-MA inhibitors. The results indicated that treatment with the proteasome inhibitor MG-132 for both 2 and 5 h significantly hindered the degradation of CfCpy*-GFP, whereas treatment with the 3-MA inhibitor did not affect the degradation of CfCpy*-GFP (Fig. 4a, b). This suggests that CfCpy*-GFP is degraded via the 26S proteasome pathway rather than through autophagy.

Further investigation was conducted on the degradation of CfCpy*-GFP in the Δ*Cfhac1* strain, Δ*Cfhac1/CfHAC1*^*mut intron*^ and Δ*Cfhac1/CfHAC1*^*S*^. Compared with the wild type, the ability of Δ*Cfhac1* to degrade CfCpy*-GFP was significantly decreased (Fig. 4c), indicating that the *CfHAC1* gene participates in the regulation of the degradation process of misfolded proteins. The unconventional splicing mutant strain Δ*Cfhac1/CfHAC1*^*mut intron*^ and the strain lacking the unconventional intron, Δ*Cfhac1/CfHAC1*^*S*^, also exhibited a significantly reduced ability to degrade CfCpy*-GFP (Fig. 4d, e), demonstrating that unconventional splicing of the *CfHAC1* gene is essential for the degradation of misfolded proteins. The same results were obtained when we used cycloheximide (CHX) to arrest protein synthesis (Krishnan et al. 2013) and then monitored the levels of CfCpy*-GFP in the three mutants (Fig. 4f). These experimental results indicate that the *CfHAC1* gene and its unconventional splicing are involved in regulating the degradation process of endoplasmic reticulum-associated misfolded proteins.

### The *CfHAC1* regulates the ubiquitination of misfolded proteins

**Fig. 5:**
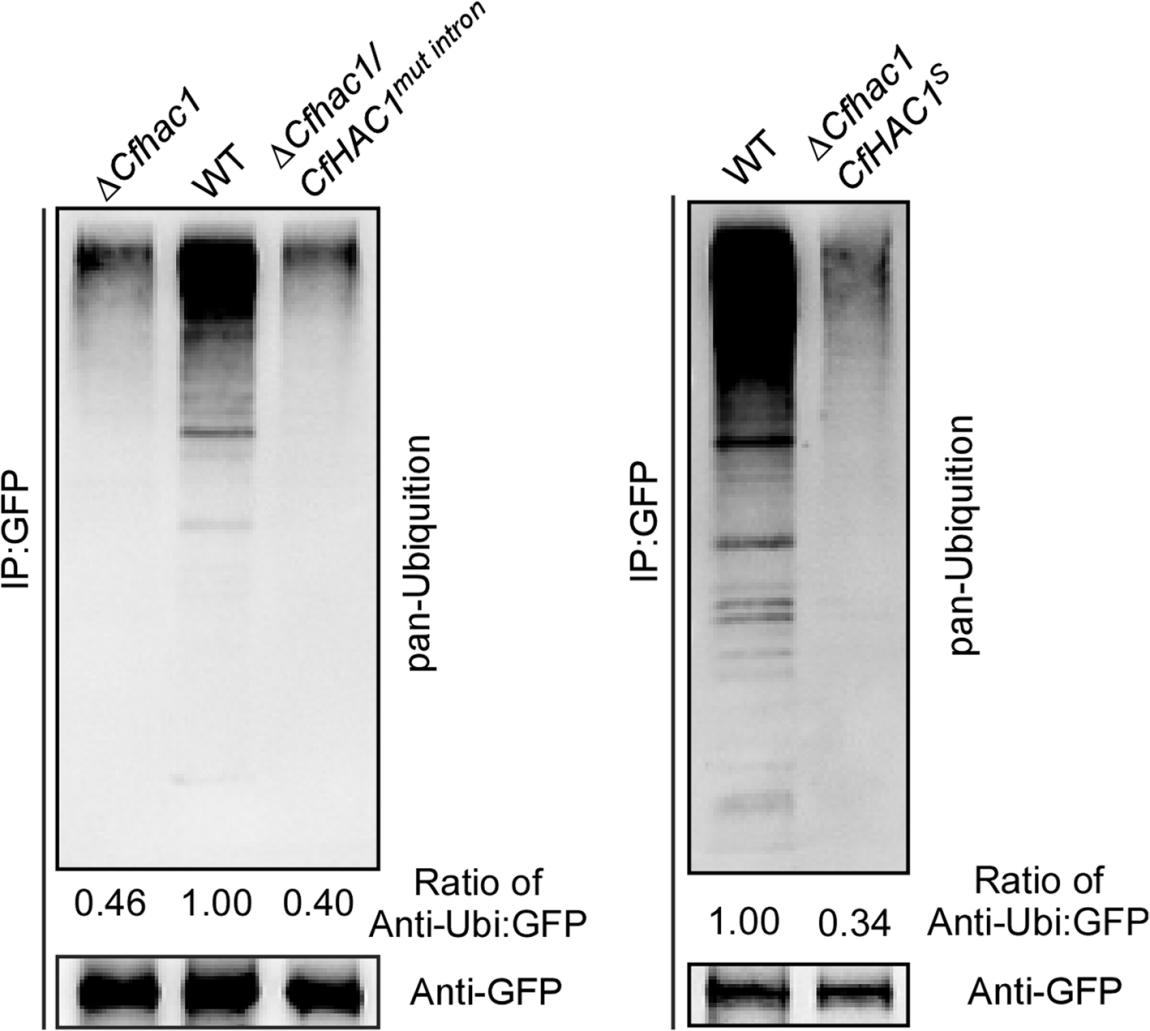
The ubiquitination of CfCpy*-GFP depends on the unconventional splicing of the *CfHAC1*. CfCpy*-GFP ubiquitination was assayed in the indicated strains by IP. The numbers were ratios of anti-ubi:GFP

Our experiments showed that the misfolded protein CfCpy*-GFP is degraded via the ubiquitin-26S proteasome pathway, influenced by the *CfHAC1* gene and its unconventional splicing. We examined the ubiquitination of CfCpy*-GFP in the Δ*Cfhac1* mutant to assess the regulatory role of *CfHAC1*. The results indicated that CfCpy*-GFP could be ubiquitinated in the wild-type strains. In contrast, there was almost no detectable ubiquitination of CfCpy*-GFP in the complete mutant Δ*Cfhac1,* Δ*Cfhac1/CfHAC1*^*mut intron*^ and the strain Δ*Cfhac1/CfHAC1*^*S*^ (Fig. 5). This lack of ubiquitination may be the primary reason for the impaired degradation of the misfolded protein CfCpy*-GFP in these mutants. The experimental results suggest that the *CfHAC1* and its unconventional splicing regulate the ubiquitination process of endoplasmic reticulum-associated misfolded proteins.

### The *CfHAC1 *affects the expression of the ubiquitin ligase genes *CfHRD1* and *CfHRD3*

In our prior investigation (LI and LI 2020), we performed a transcriptomic analysis on the Δ*Cfhac1* mutant subjected to DTT treatment, identifying 32 significantly downregulated genes enriched in the protein processing pathway within the endoplasmic reticulum. Notably, genes A09151 (XP_031893495.1) and A00730 (XP_031886770.1), which are linked to ubiquitin ligases, may play a role in the ubiquitin-26S proteasome pathway. Subsequent analysis demonstrated that the A09151 gene is composed of 2,587 base pairs, encoding 797 amino acids, and includes one RING domain along with five transmembrane domains. In contrast, the A00730 gene consists of 2,709 base pairs, encodes 842 amino acids, and contains ten SEL1 domains and a single transmembrane domain (Figure S3a). Phylogenetic analysis demonstrated that A09151 shares high homology with the E3 ubiquitin ligase Hrd1 from other fungi, clustering together with very high support (Figure S3b), particularly grouping with Hrd1 from *Colletotrichum aenigma* with 100% bootstrap support. A00730 clustered together with high support with the ubiquitin ligase Hrd3, notably with *Fusarium langsethiae*, also with 100% bootstrap support (Figure S3b). These results indicate that both genes are related to ubiquitin ligases; therefore, we designated the A09151 gene as *CfHRD1* and the A00730 gene as *CfHRD*3.

**Fig. 6:**
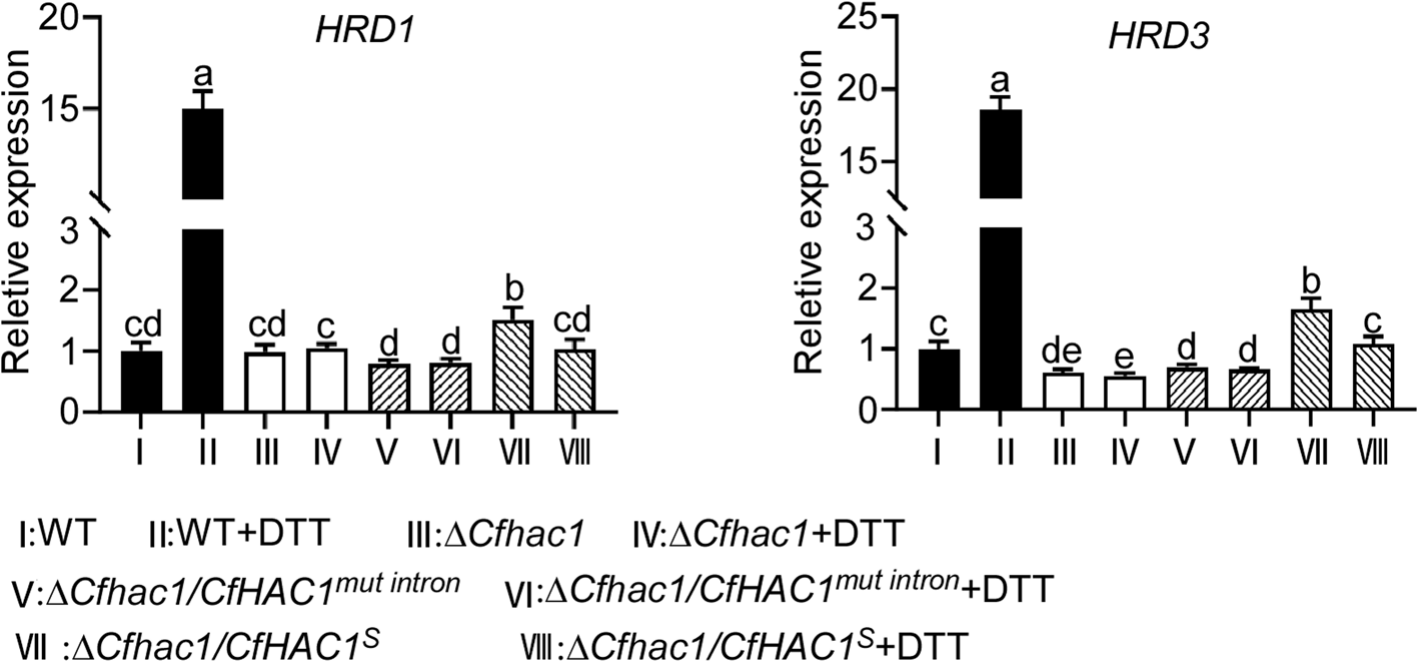
CfHac1 affects the expression of *CfHRD1* and *CfHRD3* genes. **a** Expression analysis of *CfHRD1* in the Δ*Cfhac1* mutant and wild-type strains, with or without dithiothreitol treatment. **b** Expression analysis of *CfHRD3* in the Δ*Cfhac1* mutant and wild-type strains, with or without dithiothreitol treatment. Δ*Cfhac1/CfHAC1*^*mut intron*^ represents the non-spliced CfHac1, and Δ*Cfhac1/CfHAC1*.^*S*^ represents the spliced CfHac1. Error bars represent ± SD, and different lowercase letters represent significant differences (*P* < 0.01)

We further employed qRT-PCR to validate whether the expression of the ubiquitin ligase genes *CfHRD1* and *CfHRD3* in *C. fructicola* is affected by *CfHAC1*. The results indicated that, in DTT-treated wild-type strains, the *CfHRD1* and *CfHRD3* genes were significantly upregulated. Conversely, in the mutants Δ*Cfhac1* and Δ*Cfhac1/CfHAC1*^*mut intron*^, neither DTT treatment nor the absence of DTT treatment led to the upregulation of the *CfHRD1* and *CfHRD3* genes (Fig. 6), indicating that the upregulation of these genes during endoplasmic reticulum stress is dependent on the *CfHAC1* gene and its unconventional splicing. Additionally, the upregulation of the *CfHRD1* gene in the Δ*Cfhac1/CfHAC1*^*S*^ strain was independent of DTT treatment, suggesting that the protein encoded by the artificially deleted 20 bp unconventional splicing region is capable of upregulating this gene's expression.

### *CfHRD1* and *CfHRD3* genes regulate ubiquitination-mediated degradation of misfolded proteins and pathogenicity

**Fig. 7:**
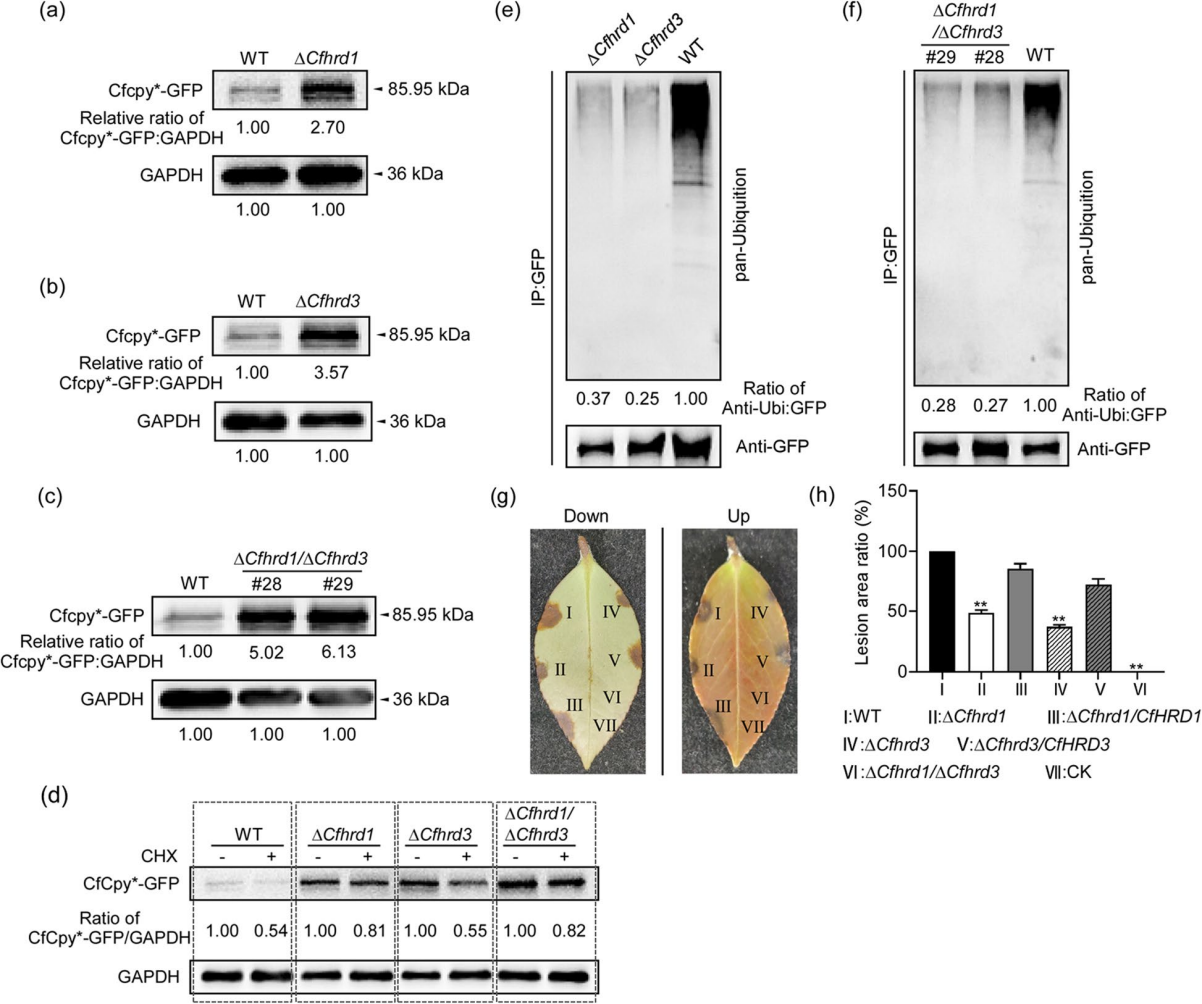
The *CfHRD1* and *CfHRD3* genes regulate the ubiquitination and degradation of CfCpy*-GFP and pathogenicity. **a** Degradation of CfCpy*-GFP in the Δ*Cfhrd1*. GAPDH was used as the control. **b** Degradation of CfCpy*-GFP in the Δ*Cfhrd3*. **c** Degradation of CfCpy*-GFP in the double knockout mutant Δ*Cfhrd1*/Δ*Cfhrd3*. **d** Degradation of CfCpy*-GFP in the three mutants following cycloheximide (CHX) treatment. **e** The ubiquitination of CfCpy*-GFP was assayed in the Δ*Cfhrd1* and Δ*Cfhrd3* strains using immunoprecipitation. **f** The ubiquitination of CfCpy*-GFP was assayed in the Δ*Cfhrd1*/Δ*Cfhrd3* using immunoprecipitation. #28, #29: two double knockout mutants. **g** Diseased symptoms of unwounded *Camellia oleifera* leaves inoculated with related mycelial blocks at 28℃ for 3 days. **h** Statistical analysis of the ratio of lesion areas of different strains. Error bars represent ± SD, and double asterisk (**) indicates significant differences (*P* < 0.01)

To elucidate the mechanisms by which the *CfHRD1* and *CfHRD3* genes respond to endoplasmic reticulum stress, we performed knockout and complementation studies on these genes (Zhang et al. 2022). The gene knockout strategy, mutant selection, and validation of complemented strains are presented in Figure S4. We further investigated the ubiquitination and degradation of the misfolded protein CfCpy*-GFP in the knockout mutants Δ*Cfhrd1* and Δ*Cfhrd3*, as well as in the double knockout mutant Δ*Cfhrd1/*Δ*Cfhrd3*. The degradation of CfCpy*-GFP was assessed after culturing the wild-type and mutant strains for 36 h. Western blot analysis revealed that, regardless of whether cycloheximide was used to arrest protein synthesis, the accumulation of the misfolded protein CfCpy*-GFP was significantly higher in the Δ*Cfhrd1* and Δ*Cfhrd3* mutants, as well as in the double knockout mutant Δ*Cfhrd1*/Δ*Cfhrd3*, compared to the wild-type (Fig. 7a, b, c, d). Notably, the accumulation of CfCpy*-GFP in the double knockout mutant was significantly greater than in the single gene knockout mutants. These results indicate that the *CfHRD1* and *CfHRD3* genes jointly regulate the degradation of endoplasmic reticulum-associated misfolded proteins.

To further investigate the mechanism by which the *CfHRD1* and *CfHRD3* genes regulate the degradation of endoplasmic reticulum misfolded proteins, we examined the ubiquitination of the misfolded protein CfCpy*-GFP in the Δ*Cfhrd1*, Δ*Cfhrd3*, and the double knockout mutant Δ*Cfhrd1*/Δ*Cfhrd3*. The wild-type and mutant strains were treated with MG-132 for 5 h, following 36 h of culture. Proteins were then extracted for co-immunoprecipitation (CoIP) using GFP beads to pull down CfCpy*-GFP, followed by detection of ubiquitination levels using ubiquitin antibodies. The results showed that the ubiquitination levels of CfCpy*-GFP were significantly reduced in the Δ*Cfhrd1*, Δ*Cfhrd3*, and the double knockout mutant Δ*Cfhrd1/*Δ*Cfhrd3*, compared to the wild-type strain (Fig. 7d, e). This reduction may be the primary reason for the impaired degradation of misfolded proteins in the mutants. In summary, the *CfHRD1* and *CfHRD3* genes mediate the degradation of endoplasmic reticulum-associated misfolded proteins by jointly regulating ubiquitination levels.

The knockout of the *CfHRD1* and *CfHRD3* genes affected the ubiquitination and degradation of misfolded proteins in *C. fructicola*; however, it remains unclear whether this impact extends to pathogenicity. We tested the pathogenicity of the Δ*Cfhrd1* and Δ*Cfhrd3* mutants, as well as the double knockout mutant Δ*Cfhrd1*/Δ*Cfhrd3*, on uninjured *Ca. oleifera* leaves. The results indicated that the pathogenicity of the Δ*Cfhrd1* and Δ*Cfhrd3* mutants was significantly reduced compared to those of the wild-type and complemented strains, with the double knockout mutant Δ*Cfhrd1*/Δ*Cfhrd3* exhibiting a complete loss of pathogenicity (Fig. 7f, g). These findings suggest that the *CfHRD1* and *CfHRD3* genes regulate the pathogenicity of *C. fructicola*.

## Discussion

In eukaryotic cells, the ER serves as a crucial site for the synthesis and maturation of secretory proteins. Misfolding and aggregation of proteins within the ER can occur due to transcriptional and translational failures, genetic mutations, or various stress conditions, leading to ER stress and affecting normal cellular function. In yeast, ER stress triggers unconventional splicing of the bZIP transcription factor Hac1, producing Hac1p. Hac1p then enters the nucleus to activate UPR target genes, boosting ER protein folding capacity and decreasing protein influx into the ER (Mori et al. 1998). In this study, we observed that the *CfHAC1* mRNA in *C. fructicola* underwent unconventional splicing in the presence of ER stressors, excising a 20 bp unconventional intron that encodes a transcriptional activator protein comprising 415 amino acids. Moreover, during the pathogen's infection of the host, the *CfHAC1* gene also exhibited unconventional splicing, indicating that the pathogen experienced ER stress during its interaction with the host. Further investigation revealed that the transcriptional activator protein produced through this unconventional splicing entered the nucleus under ER stress and upregulated the expression of UPR target genes *CfPDI1*, *CfLHS1*, *CfKAR2*, *CfSIL1*, and *CfSCJ1*. This suggests that this transcription factor functions comparably across different fungi and similarly regulates the UPR process.

Furthermore, the mutant strain Δ*Cfhac1/CfHAC1*^*mut intron*^, which was obtained through multiple site mutations in the intron region and is incapable of normal splicing, fails to form appressoria and does not produce lesions on either wounded or unwounded tea leaves. This finding contrasts with the results from Tang et al. (2015), where the non-splicing mutant was able to form appressoria, indicating a certain degree of functional divergence of the *HAC1* gene among different species. The mechanism by which the constitutively activated UPR mutant Δ*Cfhac1/CfHAC1*^*S*^ results in smaller lesions on wounded leaves and no lesions on unwounded leaves requires additional study. In summary, this study reveals that the *CfHAC1* gene requires unconventional splicing to regulate mycelial growth, conidiation, appressorium formation, the response to ER stress, and pathogenicity.

The ER is a crucial organelle responsible for protein folding and transport. Protein folding stress within the ER leads to the accumulation of unfolded or misfolded proteins. In eukaryotic cells, there are two main pathways for regulating ER stress. The first is the unfolded protein response, which enhances protein folding capacity by upregulating the expression of molecular chaperones, which assist in proper protein folding, and foldases, which facilitate the folding process. The second is the endoplasmic reticulum-associated protein degradation pathway, which exports substrates that cannot be correctly folded from the ER for degradation by the cytoplasmic ubiquitin–proteasome system (Smith et al. 2011). These two pathways are essential quality control systems that enable cells to respond to environmental stress, regulate ER folding capacity, and maintain dynamic equilibrium within the ER. The carboxypeptidase Y mutant Cpy*, which is prone to misfolding and degradation within the ER, is often used as a model protein in studies of ER-associated misfolded protein degradation (Hiller et al. 1996; Vashistha et al. 2016). In this study, we constructed the carboxypeptidase Y mutant CfCpy* in the pathogenic fungus *C. fructicola* and confirmed that CfCpy* is degraded via the ubiquitin-26S proteasome pathway rather than through autophagy.

In the cytosol, the unconventional splicing of *HAC1* mRNA is mediated by Ire1. In mammals, IRE1α, recognized as a sensor of the unfolded protein response, has been definitively identified as a substrate of the Hrd1 ER-associated degradation complex. Under basal conditions, IRE1α is targeted for degradation via the ERAD pathway. However, during endoplasmic reticulum stress, IRE1α dissociates from the ERAD complex, leading to its stabilization. This process underscores ERAD-mediated turnover of IRE1α as an intrinsic self-regulatory mechanism within the endoplasmic reticulum quality control systems (Sun et al. 2015). In fungal pathogens, the simultaneous loss of *Aspergillus fumigatus* ERAD component derA and UPR regulator hacA severely impacts hyphal growth, antifungal resistance, protease secretion, and virulence more than the loss of either gene alone, indicating DerA's cooperation with the UPR in promoting *A. fumigatus* virulence (Richie et al. 2011). This experiment utilized CfCpy* as a misfolded protein to investigate the role of the *CfHAC1* gene in the degradation of ER-associated misfolded proteins in the pathogenic fungus. The study found that the ability to degrade CfCpy* decreased upon deletion of the *CfHAC1* or inability to undergo unconventional splicing. Ubiquitination assays demonstrated that the levels of CfCpy* ubiquitination were reduced in the Δ*Cfhac1* and Δ*Cfhac1/CfHAC1*^*mut intron*^ mutants, suggesting that this reduction is a primary reason for impaired CfCpy* degradation in the mutants. In summary, we propose that the bZIP transcription factor CfHac1, along with its unconventional splicing, regulates the ubiquitination of ER-associated misfolded proteins, thereby influencing their degradation process. Thus, the transcription factor CfHac1 not only regulates the UPR pathway but also plays a significant role in modulating the ERAD pathway.

**Fig. 8:**
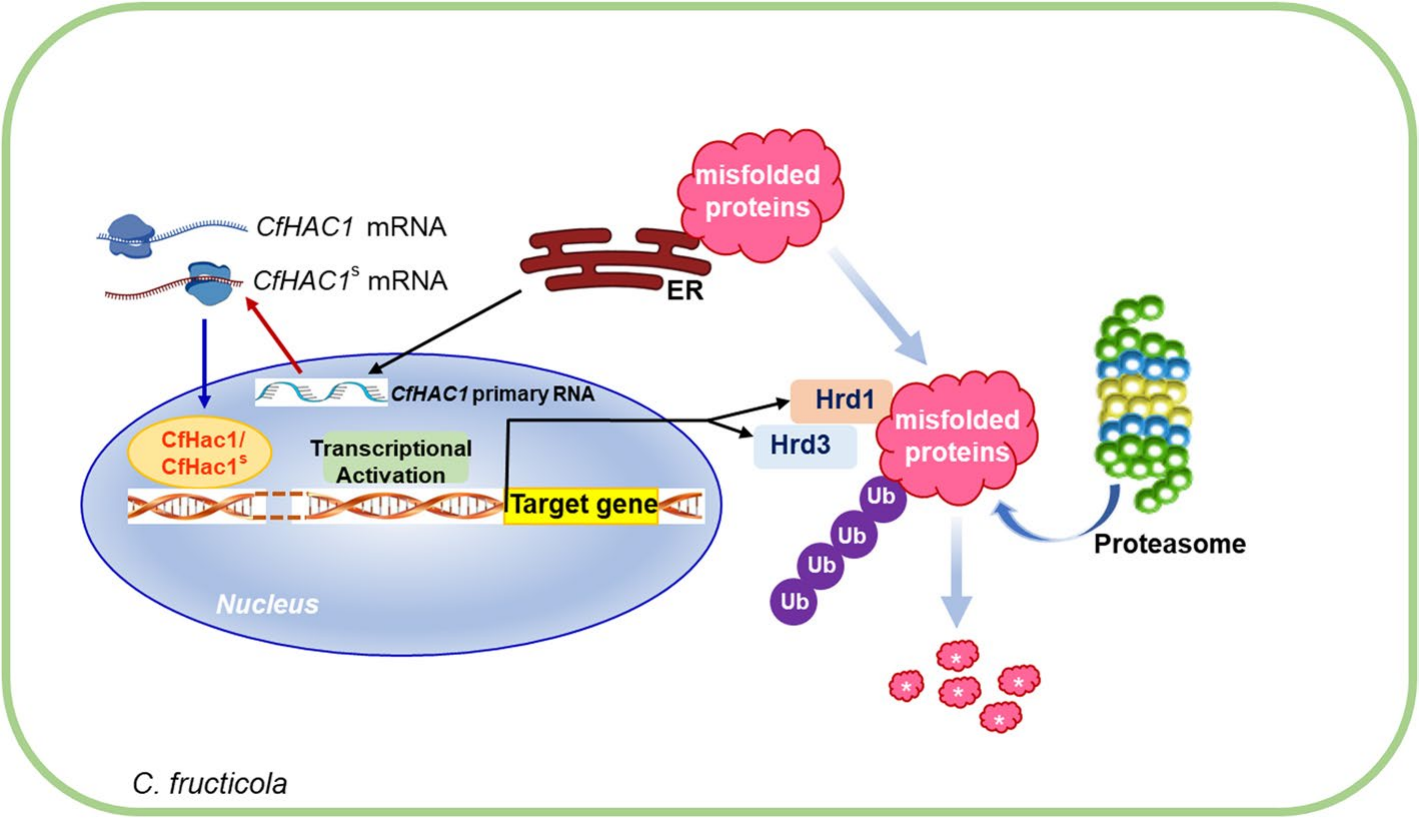
A working model of Cfhac1 regulates the ERAD pathway in *Colletotrichum fructicola*. White asterisk indicates degraded misfolded proteins. ER, endoplasmic reticulum. Ub, ubiquitination

The HRD (HMG-CoA reductase degradation) complex, a key component of ERAD, mainly consists of Hrd1, Hrd3, and the lectin Yos9, and is responsible for the recognition, transport, and ubiquitination of misfolded proteins within the endoplasmic reticulum. Hrd3 serves as a component of the Hrd1 complex within the ERAD pathway, further regulating substrate recognition and maintaining the stability of the Hrd1 protein through its interaction with it (Vashistha et al. 2016). Carvalho et al. (2010) discovered that substrates are transferred from Hrd3 to Hrd1, and Hrd3 can modulate the function of Hrd1. Mehnert et al. (2014) found that substrates within the endoplasmic reticulum lumen bind to Yos9 and Hrd3 before being passed to Der1, subsequently interacting with Hrd1 to further localize the substrate to the Hrd1 complex. In eukaryotes, the absence of Hrd1 results in a failure to degrade ERAD substrates in a timely manner (Carvalho et al. 2010; Krishnan et al. 2013; Vashistha et al. 2016; Yokota et al. 2017). In this study, the deletion of the *CfHRD1* and *CfHRD3* resulted in a decrease in the ubiquitination level of CfCpy* and obstructed its degradation, indicating that the E3 ubiquitin ligase genes *CfHRD1* and *CfHRD3* regulate the degradation of CfCpy* through its ubiquitination. Our research also revealed that in the Δ*Cfhac1* and non-splicing mutant Δ*Cfhac1/CfHAC1*^*mut intron*^, the ubiquitination level of CfCpy* was significantly reduced, leading to impaired degradation. Additionally, we observed that in all *CfHAC1*-related mutants under endoplasmic reticulum stress, the expression of *CfHRD1* and *CfHRD3* were not upregulated, whereas both genes were significantly upregulated in the wild-type strain. Based on these findings, we hypothesize that the inability of the Δ*Cfhac1* mutant to degrade CfCpy* in a timely manner is due to the failure to upregulate the expression of *CfHRD1* and *CfHRD3*, which affects their ubiquitination. Collectively, these results indicate that under endoplasmic reticulum stress, the *CfHAC1* modulates the upregulation of the E3 ubiquitin ligase genes *CfHRD1*, and its component *CfHRD3*, through unconventional splicing, leading to the ubiquitination of endoplasmic reticulum-associated misfolded proteins and thereby facilitating their degradation. Accordingly, we propose a working model of the transcription factor Cfhac1 in regulating the ERAD pathway in *C. fructicola*. The accumulation of misfolded proteins induces endoplasmic reticulum stress, activating the *CfHAC1* to undergo unconventional splicing. This process the transcriptional activator Cfhac1^S^, which subsequently enters the nucleus to regulate the expression of the E3 ubiquitin ligase genes *CfHRD1* and *CfHRD*3. These ligases ubiquitinate endoplasmic reticulum-associated misfolded proteins, mediating their degradation and alleviating cellular stress (Fig. 8).

Our research findings indicate that, in the process of infecting onions, the *CfHAC1* undergoes unconventional splicing, suggesting that the pathogen experiences endoplasmic reticulum stress during host infection. This stress may arise from the necessity for the pathogen to secrete a substantial amount of extracellular enzymes to invade plant cells, leading to increased endogenous endoplasmic reticulum stress due to the large synthesis of secreted proteins. Additionally, the host's defensive response may impose exogenous endoplasmic reticulum stress on the pathogen. Our studies have shown that the pathogenicity of the Δ*Cfhrd1* and Δ*Cfhrd3* mutants was significantly reduced, with the double knockout mutant, Δ*Cfhrd1*/Δ*Cfhrd3*, completely losing its pathogenicity. We speculate that these three types of mutants experience increased endoplasmic reticulum stress due to the impeded degradation of misfolded proteins, which results in diminished pathogenicity during their interaction with the host. Similar findings have been reported in other plant pathogens. Yi et al. found that the absence of the endoplasmic reticulum chaperone MoLhs1 leads to a continuously activated UPR signaling pathway, disrupting endoplasmic reticulum homeostasis and reducing the strain's pathogenic capacity (Yi et al. 2009). Tang et al. discovered that *MoHRD1* not only participates in the degradation of misfolded proteins but also regulates the pathogenic process in the rice blast fungus (Tang et al. 2020).

In summary, the results of this study significantly elucidate the pathogenic mechanisms mediated by the transcription factor CfHac1 in regulating the ERAD pathway. Additionally, they provide a theoretical basis for the development of novel agents targeting key genes within this pathway.

## Materials and methods

### Strains

In this study, the wild-type strain of *C. fructicola*, CFLH16, was isolated and preserved in our laboratory. The *CfHAC1* gene knockout mutant strain, Δ*Cfhac1*, and the complemented strain, Δ*Cfhac1/CfHAC1*, were obtained from previous research conducted in our laboratory (LI and LI 2020; Yao et al. 2019).

### Extraction of DNA, RNA, and synthesis of cDNA

The specific methods were based on those described by Tang et al. (2015). For RNA extraction at the infection stage, onion epidermis was point-inoculated with droplets of conidial suspension (1x10^6^) of the wild-type strain of *C. fructicola*, and maintained in the dark at 28 °C with high humidity for 36 to 48 h. Tissues were collected, ground into a powder using a mortar and pestle for RNA extraction, and then reverse transcribed into cDNA.

### Observation of CfHac1 subcellular localization

The GFP protein was fused to the N-terminus of CfHac1 and transformed into the Δ*Cfhac1* mutant. The resulting green fluorescent transformants, which restored the phenotype, were examined for localization. The hyphae of these transformants were collected and treated with 10 mM dithiothreitol (DTT) for 1 h, followed by DAPI counterstaining, with untreated samples serving as controls. Changes in subcellular localization of GFP were observed using a fluorescence microscope.

### *CfHAC1* mRNA unconventional splicing site and mutation analysis

The wild-type CFLH16 strain was cultured in liquid media for 36 h and then treated with 10 mM DTT for 1 h, using untreated samples as controls. Total RNA was extracted from the strain, and the first strand of cDNA was synthesized through reverse transcription. Primers were designed (hac1-atgF/hac1-tgaR) for the PCR amplification of the *CfHAC1* coding region at both ends, followed by TA cloning and sequencing to compare sequence differences (Figure S1).

Obtain strains with mutations at unconventional splicing sites using in situ complementation. This strategy is illustrated in Figure S2a. Using codon degeneracy, unconventional splicing site mutation primers bZIP13-1 F/hac1 s-R1 and hac1 s-F2/bZIP13-4R were designed to PCR amplify approximately 1000 bp fragments of the *CfHAC1* and its flanking arms from the wild-type strain (Figure S2b, c). These were fused with a G418 resistance gene and replaced the hygromycin phosphotransferase gene (*HPH*) in the *CfHAC1* knockout mutant Δ*Cfhac1* via homologous recombination, resulting in the mutant strain Δ*Cfhac1/CfHAC1*^*mut intron*^. Using primers bZIP13-1 F/Quqie-R1 and Quqie-F2/bZIP13-4R, a similar method was employed to obtain the mutant strain Δ*Cfhac1/CfHAC1*^S^, which had a deletion of a 20 bp unconventional splicing region (Figure S2 d).

RT-PCR was employed to analyze the transcription of the *CfHAC1* gene in C. fructicola during treatment with the endoplasmic reticulum stress inducer DTT and during infection of onions. cDNA was amplified using semi-quantitative PCR primers *HAC1*−54F/*HAC1*−55R from the pathogen during infection and subjected to low-voltage electrophoresis at 80 V on a 3% agarose gel.

### Sensitivity assessment of mutants to endoplasmic reticulum stress agents

Colony edges of the tested strains were sampled using a cork borer (Φ = 6 mm) and inoculated onto PDA medium containing 5 mM DTT, with PDA media without DTT serving as the control. The samples were incubated in the dark at 28 °C for 3 days, after which images were taken, and the inhibition rates were calculated. Each strain was tested in triplicate, and the experiment was repeated three times.

### Assessment of hyphal growth and pathogenicity of mutants

A cork borer was used to cut out a mycelial disc (Φ = 6 mm) from the edge of the tested strain's colony, which was then inoculated onto PDA, CM and MM media. After incubation in the dark at 28 °C for 3 days, the colony diameter was measured. Additionally, a mycelial disc (Φ = 8 mm) was cut from the edge of the colony and inoculated onto detached oil tea leaves using both wounded and non-wounded methods, with sterile PDA media serving as a blank control. The samples were incubated for 2 to 4 days, and the size of the lesions on the leaves was compared. The lesions on inoculated leaves were photographed and statistical analysis of lesion sizes measured by Image J.

### Quantitative real-time PCR for gene expression detection

Total RNA was extracted from the mycelium and reverse transcribed to synthesize the first strand of cDNA. *ACTIN* (XM_007276777) was used as the internal reference gene, and homologous prediction of UPR target genes reported in yeast was conducted in the genome of *C. fructicola*. Primers (Table S1) were designed based on their coding sequences to detect the expression levels of these genes in the tested strains. RT‑qPCR was performed using the ChamQ SYBR qPCR Master Mix kit (Vazyme, Q331) on an ABI Quant Studio 3 platform. The reaction program consisted of: 95 °C for 30 s; 95 °C for 10 s and 60 °C for 30 s for 40 cycles; 95 °C for 15 s, 60 °C for 1 min, and 95 °C for 15 s. The experiment was conducted in triplicate, with three repeats for each run.

### Construction of the CfCpy*-GFP plasmid and degradation assay

The plasmid of misfolded protein CfCpy*-GFP was constructed according to the method of reference (Krishnan et al. 2013; Tang et al. 2020). Through site-directed mutagenesis, glycine at position 269 of the carboxypeptidase Y from *C. fructicola* was mutated to alanine (G269 A), constructing a plasmid vector for the misfolded protein CfCpy*-GFP driven by a strong promoter (RP27). The plasmid was transformed into the wild-type and mutant strains using a protoplast transformation method. Total proteins were extracted, and western blotting was performed to detect the degradation of CfCpy*-GFP.

### Detection of ubiquitination levels of CfCpy*-GFP

The wild-type and mutant strains were cultivated in liquid media for 36 h and then treated with MG-132 for 5 h, after which total proteins were extracted. Immunoprecipitation (Co-IP) of the extracted total proteins was conducted using GFP-modified agarose beads (GFP-agarose beads), and Western blotting was performed to assess the ubiquitination status of CfCpy*-GFP.

### Gene deletion and complementation

The *CfHRD1* and *CfHRD3* gene deletions were performed using the standard one-step strategy (Li et al. 2021b; Luo et al. 2023; Zhang et al. 2022). The strategy for the deletion of *CfHRD1* and *CfHRD3* is illustrated in Supporting Information Figure S4a. First, we overlapped the two approximately 1.0 kb sequences flanking *CfHRD1* and *CfHRD3* on either side of the *HPH* gene. Subsequently, we amplified and introduced the resulting approximately 3.4 kb fragments, which included the flanking sequences and the hygromycin resistance cassette, into the protoplasts of the wild-type strain (Figure S4b, c). Third, gene *CfHRD3* was knocked out in mutant Δ*Cfhrd1* to create the double knockout mutant (Figure S4 d). Finally, the complementary fragments were amplified by PCR using primers and inserted into pYF11 (which confers bleomycin resistance) to complement the mutant strain. All primers used are listed in Table S1.

### Statistical analysis

All data were presented as mean ± standard deviation and analyzed using a one-way analysis of variance (ANOVA) followed by Duncan’s new multiple range test. A *p*-value of < 0.01 or 0.05 was considered to represent a significant difference.

## Supplementary Information

Supplementary Material 1.

## Data Availability

The datasets analyzed during the current study are available from the corresponding author upon reasonable request.

## Abbreviations

ER: Endoplasmic reticulum
UPR: Unfolded protein response
bZIP: The basic leucine zipper
Ire1: Inositol-requiring enzyme 1
ERAD: Endoplasmic reticulum-associated protein degradation
Ub: Ubiquitin
UPS: Ubiquitin-proteasome system
E1: Ubiquitin-activating enzyme
E2: Ubiquitin-conjugating enzyme
E3: Ubiquitin ligase
DTT: Dithiothreitol
HPH: Hygromycin B resistance gene
RT‑qPCR: Reverse Transcription Quantitative Real-time Polymerase ChainReaction
Co-IP: Immunoprecipitation
ANOVA: One-way analysis of variance
MG-132: Carbobenzoxy-L-leucyl-L-leucyl-L-leucinal
3-MA: 3-Methyladenine, a phosphoinositide 3-kinase inhibitor
PI3 K: Phosphoinositide 3-kinase
CPY*: Carboxypeptidase Y mutant
HRD: HMG-CoA reductase degradation
DAPI: 4',6-Diamidino-2-phenylindole
GAPDH: Glyceraldehyde-3-phosphate dehydrogenase
kDa: Kilodaltons
WT: Wild type
PDA: Potato dextrose agar
CM: Complete medium
MM: Minimal medium
PCR: Polymerase Chain Reaction
IP: Immunoprecipitation
GFP: Green Fluorescent Protein
