# Supplementary material for: The transcription factor CfHac1 regulates the degradation of ubiquitin-mediated ER-associated misfolded proteins and pathogenicity in Colletotrichum fructicola
Source: Stress Biol. 2025 Jun 12;5(1):41. doi: 10.1007/s44154-025-00237-6 (PMC12162442; doi:10.1007/s44154-025-00237-6)
Supplement: Supplementary file 1 — Supplementary Material 1. [file 44154_2025_237_MOESM1_ESM.docx]

**Supplementary materials**

**Table S1. Primers used for PCR and plasmid construction.**

| Primers | Sequences （5` to 3`） | Applications |
| --- | --- | --- |
| hac1-atgF | ATGGCTGCTTGGGAACAGAC | Amplification of the *CfHAC1* coding region |
| hac1-tgaR | AGCTAATCGACAACGCTTCC | Amplification of the *CfHAC1* coding region |
| bZIP13-1F | AACCATGGAGTCGCTACACG | Amplify the 5' flank sequence for point mutations in the unconventional splicing region of *CfHAC1* |
| bZIP13-4R | TGCACCAGCTTCTATCACAC | Amplify the 3' flank sequence for point mutations in the unconventional splicing region of *CfHAC1* |
| bZIP13-7F | TCCTCCGACTTGACACAACG | Validation of *CfHAC1* delation |
| bZIP13-8R | ACGTCCGCTTCATCACACTT | Validation of *CfHAC1* delation |
| H855R | GCTGATCTGACCAGTTGC | Validation of targeted-gene delation |
| HAC1G418UR | CAATATCATCTTCTGTCGACTCAAGCTAATCGACAACGCT | Amplify the 5' flank sequence for point mutations in the unconventional splicing region of *CfHAC1* |
| HAC1G418DF | TTCTTGACGAGTTCTTCTGATAAAGGTTATGACTATGGTG | Amplify the 3' flank sequence for point mutations in the unconventional splicing region of *CfHAC1* |
| *HAC1*-54F | CGCAGAAGATGACGAGGCAG | RT-PCR for *CfHAC1* spliced sequence |
| *HAC1*-55R | GAGAGGCCAGCCATGATTGC | RT-PCR for *CfHAC1* spliced sequence |
| Hac1S-R1 | AAGATCGCAGAGCATTGCACTAGGACGTTGTGTCAAGTCGG | Construction of *CfHAC*1^mut intron^ |
| Hac1S-F2 | AGTGCAATGCTCTGCGATCTTCAGTGTCAACAGTCGGAGGA | Construction of *CfHAC*1^mut intron^ |
| QuqieR1 | CAGGACGTTGTGTCAAGTCG | Construction of *CfHAC*1^S^ |
| QuqieF2 | CGACTTGACACAACGTCCTGCAGTGTCAACAGTCGGAGGAC | Construction of *CfHAC*1^S^ |
| A00730-1F | GTGACTGAGTTGCTGCGACT | Amplify *CfHRD3* 5' flank sequence |
| A00730-2R | TTGACCTCCACTAGCTCCAGCCAAGCCGCTGCGAGTATTATGTGCAA | Amplify *CfHRD3* 5' flank sequence |
| A00730-3F | CAAAGGAATAGAGTAGATGCCGACCGGCTGACATGTATGATACGAA | Amplify *CfHRD3* 3' flank sequence |
| A00730-4R | CGATACTTGCTGCAGAAGCT | Amplify *CfHRD3* 3' flank sequence |
| A00730-5F | TGTCATAGACGTTGACGAGG | Validation of *CfHRD3* delation |
| A00730-7F | TGGGTTGTTGAGTCGTACCG | Validation of *CfHRD3* delation |
| A00730-8R | GCAGCCTTGAAGAGGTCAGT | Validation of *CfHRD3* delation |
| A00730-9F | ACTCACTATAGGGCGAATTGGGTACTCAAATTGGTTCAACCTTGGTAGTGACGTTG | Construction of *CfHRD3* complementation |
| A00730-10R | CACCACCCCGGTGAACAGCTCCTCGCCCTTGCTCACGTGACCAATGCCTCCCGCGA | Construction of *CfHRD3* complementation |
| A00730-QRTF | GGCTTGGCGTCAACAACAAT | qRT-PCR primer of *CfHRD3* |
| A00730-QRTR | CCAGGTGGAAGTCCTGTGTC | qRT-PCR primer of *CfHRD3* |
| A09151-UF | GTGTAGCGTGGACATCTTGA | Amplify *CfHRD1* 5' flank sequence |
| A09151-G418UR | CAATATCATCTTCTGTCGACCTTCGCGTTCGATGGGCTGA | Amplify *CfHRD1* 5' flank sequence |
| A09151-G418DF | TTCTTGACGAGTTCTTCTGAACGATGCGGGACAGTAGGCA | Amplify *CfHRD1* 3' flank sequence |
| G418YZR | TGACCAGTTGCCTAAATGAA | Validation of *CfHRD1* delation |
| A09151-DR | ACTGGGTATGTGTACATGGA | Amplify *CfHRD1* 3' flank sequence |
| A09151-7F | AGCGAACTTTTACTCGGCCA | Validation of *CfHRD1* delation |
| A09151-8R | CGCAGTCTAGAGGGTTAGCG | Validation of *CfHRD1* delation |
| A09151-9F | ACTCACTATAGGGCGAATTGGGTACTCAAATTGGTTGAGCAATCGTTTCGACGTCC | Construction of *CfHRD1* complementation |
| A09151-10R | CACCACCCCGGTGAACAGCTCCTCGCCCTTGCTCACCTCATCGTCCTCGGCCTCCT | Construction of *CfHRD1* complementation |
| A09151-QRTF | GTTCCGATCCACCCAACACT | qRT-PCR primer of *CfHRD1* |
| A09151-QRTR | TGTGTGGATGCAGGGACTTC | qRT-PCR primer of *CfHRD1* |
| PDI1-QF | GAAGTTCCCCTTCGACCAGG | qRT-PCR primer of *CfPDI1* |
| PDI1-QR | GGGCGTAGAACTCGATCAGG | qRT-PCR primer of *CfPDI1* |
| LHS1-QF | GCCGAGAAGATCCGTAGCAA | qRT-PCR primer of *CfLHS1* |
| LHS1-QR | GAGAAGTCGTCGTGGTTGGT | qRT-PCR primer of *CfLHS1* |
| KAR2-QF | ATATCGTTCTCGTCGGTGGC | qRT-PCR primer of *CfKAR21* |
| KAR2-QR | GCTTGGTCATGACACCTCCA | qRT-PCR primer of *CfKAR21* |
| SCJ1-QF | GCACGTTCTTCAGACGCAAG | qRT-PCR primer of *CfSCJ1* |
| SCJ1-QR | AGTCCACAAACAGGTTGCCA | qRT-PCR primer of *CfSCJ1* |
| SIL1-QF | GGCGGAATGTTACCCCAAGA | qRT-PCR primer of *CfSIL1* |
| SIL1-QR | CTTGGGCAGTCTCGGTTCAT | qRT-PCR primer of *CfSIL1* |
| ActQRTF | ATCAACCCCAAGTCCAACAG | qRT-PCR primer of *ACTIN* |
| ActQRTR | CGATTTCACGCTCGGCAGT | qRT-PCR primer of *ACTIN* |
| CPY-UP-F | ACTCACTATAGGGCGAATTGGGTACTCAAATTGGTTCAGGCTTGGCAGCCGGTACC | Amplify a 1.5 kb promoter region of *CfCPY* gene for constructing “pYF11-Native-promoter-NEGFP-*CfCPY*” plasmid |
| CPY-UP-R | TCCTCGCCCTTGCTCACCATGGTGTGGGCAGGCAATTAGC |  |
| EGFP-F | ATGGTGAGCAAGGGCGAGGA | Amplify a 0.7 kb *EGFP* gene without termination codon |
| EGFP-R | CTTGTACAGCTCGTCCATGC |  |
| CPY-ATG-F | GCATGGACGAGCTGTACAAGATGAGGTTCTCAACCTCCGC | Amplify a 0.8 kb upstream fragment of position 269 of CfCPY (from “ATG” to “G269A”) for constructing G269A mutation |
| G269A-R | GTGGCCGGCATAGGACTCGGCAGCAATGTGGAAGTCCTGC |  |
| G269A-F | GCAGGACTTCCACATTGCTGCCGAGTCCTATGCCGGCCAC | Amplify a 0.8 kb downstream fragment of position 269 of CfCPY (from “G269A” to “TAA”) for constructing G269A mutation |
| CPY-TAA-R | CACCACCCCGGTGAACAGCTCCTCGCCCTTGCTCACTTAAGCGCTCCACTCACCGC |  |
| RP27-EGFP-F | CAGATCTTGGCTTTCGTAGGAACCCAATCTTCAATGGTGAGCAAGGGCGAGGA | Amplify a 0.7 kb *EGFP* gene (5`-terminal can be integrated with RP27 promoter) without “TAA” for constructing “pYF11-RP27 promoer-NEGFP-*CfCPY*” plasmid |
| EGFP-R | CTTGTACAGCTCGTCCATGC |  |


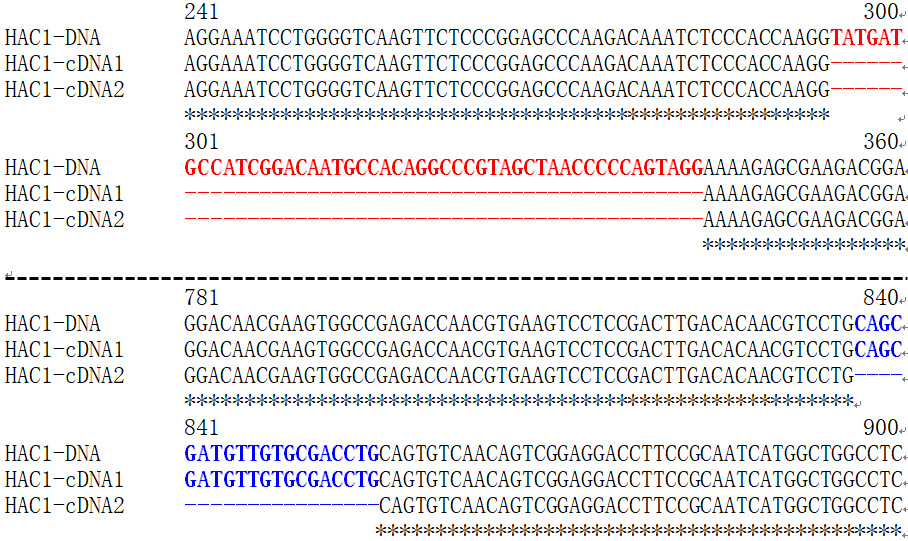


**Figure S1. Comparison of partial cDNA sequences of two transcripts of the *CfHAC1* gene.** The red part is a 49 bp (base pairs) conventional intron, and the blue part is a 20 bp unconventional intron.


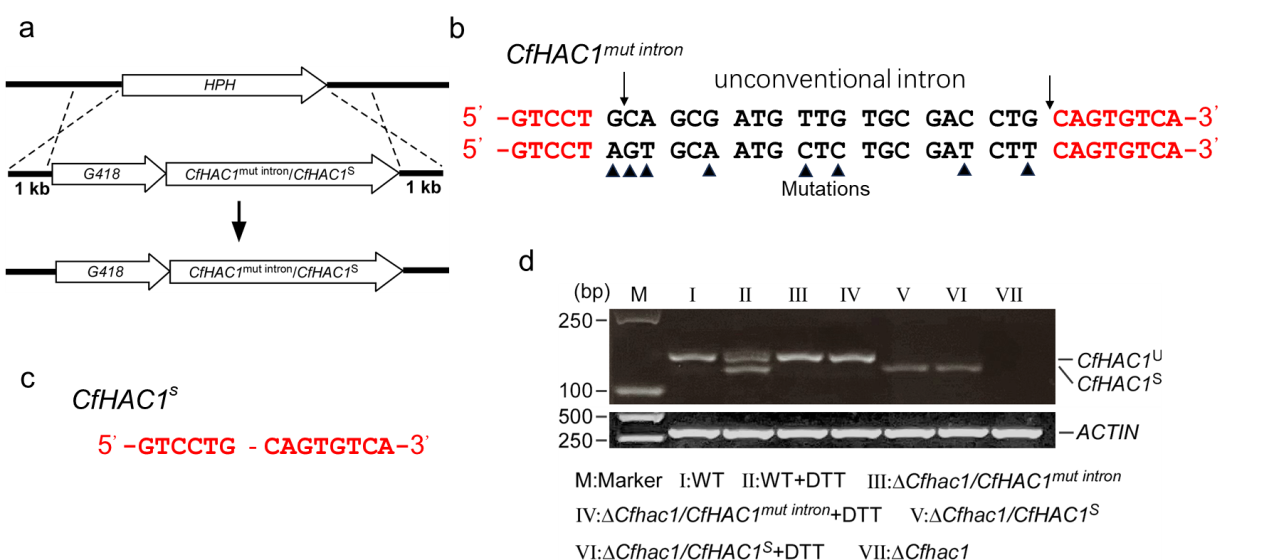


**Figure S2. Point mutations in the unconventional splicing region of the *CfHAC1* gene.** (a) Point Mutation Strategy of *CfHAC1*: Utilizing the principle of homologous recombination, point mutation strains Δ*Cfhac1/CfHAC1^mut intron^* and Δ*Cfhac1/CfHAC1^S^* were generated through in situ complementation. The introduction of point mutations was achieved via a site-directed PCR approach. (b) Eight mutations were engineered to inhibit unconventional splicing triggered by DTT, as denoted by black triangles indicating the mutated nucleotides. (c) The atypical intron was artificially excised to produce the spliced form of CfHac1. (d) RT-PCR analysis demonstrated the occurrence of unconventional splicing in the resultant transformants. Endoplasmic reticulum stress was induced by the addition of 10 mM DTT to the culture medium for 1 hour. The band labeled *CfHAC1^U^* corresponds to the transcript where unconventional splicing is absent, whereas the bands labeled *CfHAC1^S^* represent transcripts that have undergone unconventional splicing. *ACTIN* was used as the control.


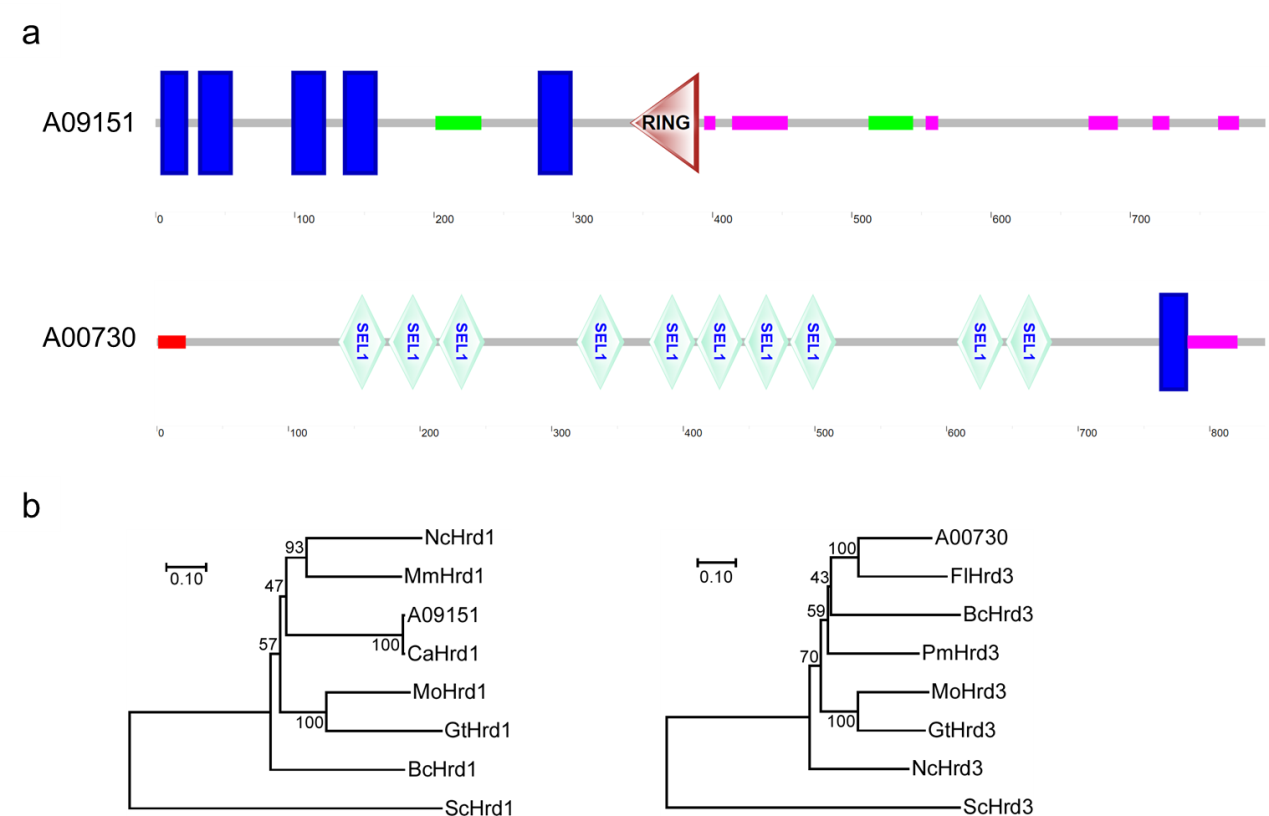


**Figure S3.** **Phylogenetic analysis and domain prediction of A09151 and A00730.** (a) The domain predictions for proteins A09151 and A00730 are illustrated, where the blue rectangle denotes the transmembrane domain, the brown triangle signifies the RING domain, and the pale green rhombus indicates the SEL1 domain. The alignment of A09151 and A00730 proteins from various fungal species was performed using CLUSTAL_W. Subsequently, the phylogenetic tree was constructed employing MEGA 7.0 and the neighbor-joining method, with 1000 bootstrap replicates to ensure statistical robustness. (b) The sequences were collected from the NCBI database and the GenBank accession numbers are shown as follows: ScHrd1: *S. cerevisiae* (NP_014630.1), MoHrd1: *M. oryza*e (XP_003709820.1), NcHrd1: *N. Crassa* (XP_959176.1), BcHrd1: *B. Cinerea* (XP_024551184.1), GtHrd1: *G. Tritic i*(XP_009226642.1), MmHrd1: *M. mycetomati*s (KXX78867.1), CaHrd1: *C. aenigma* (XP_037177623.1); ScHrd3: *S. cerevisiae* (NP_013308.1), MoHrd3: *M. oryzae* (XP_003716287.1), NcHrd3: *N. Crassa* (XP_965700.3), BcHrd3: *B. Cinerea* (XP_024548639.1), GtcHrd1: *G. Tritici* (XP_009216991.1), PmHrd1: *P. Minimum* (XP_007914455.1) , FLHrd1: *F. Langsethiae* (KPA45812.1).

**
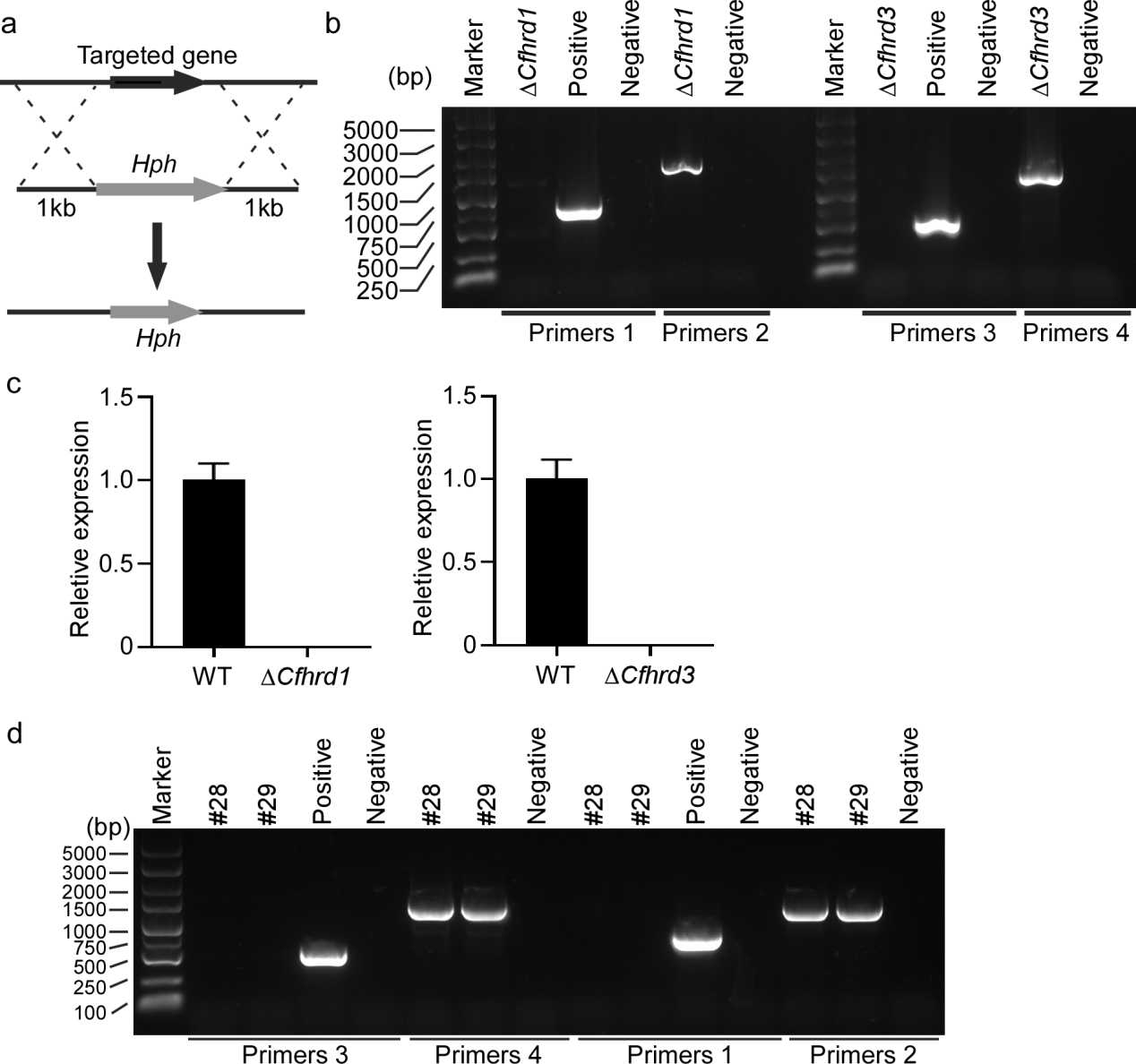
**

**Figure S4.** **Generation of *CfHRD1* and *CfHRD3* gene deletion mutants in *C. fructicola*.** (a) A

schematic representation of the deletion strategy employed for the *CfHRD1* and *CfHRD3* genes. (b) Confirmation of the gene deletion mutants through PCR amplification using primers. (c) Expression of *CfHRD1* and *CfHRD3* in the mutants *Cfhrd1* and Δ*Cfhrd3*, respectively. (d) Verification of the double-knockout mutants for both *CfHRD1* and *CfHRD3* genes using specific primers. #28 and #29 represent the double-knockout mutants. Primers 1:A09151-7F/A09151-8R; Primers 2: A09151-UF/G418YZR; Primers 3: A00730-7F/A00730-8R; Primers 4: A00730-5F/H855R.
